# Comparative Study on Lead and Copper Biosorption Using Three Bioproducts from Edible Mushrooms Residues

**DOI:** 10.3390/jof7060441

**Published:** 2021-05-31

**Authors:** Nathália R. C. M. Castanho, Renan A. de Oliveira, Bruno L. Batista, Bruna M. Freire, Camila Lange, André M. Lopes, Angela F. Jozala, Denise Grotto

**Affiliations:** 1 University of Sorocaba, Sorocaba 18023-000, SP, Brazil; nathalia.rcmc@gmail.com (N.R.C.M.C.); renan.oliveira@prof.uniso.br (R.A.d.O.); 2 Center of Natural and Human Sciences, Federal University of ABC, Santo André 09210-170, SP, Brazil; bruno.lemos@ufabc.edu.br (B.L.B.); bruna.freire@ufabc.edu.br (B.M.F.); camilosalange@yahoo.com (C.L.); 3 Faculty of Pharmaceutical Sciences, University of Campinas, Campinas 13083-871, SP, Brazil; amlopes@unicamp.br

**Keywords:** biosorption, bioremediation, *Agaricus bisporus*, *Lentinula edodes*, heavy metals, metal contamination

## Abstract

Agricultural waste products can be used as biosorbents for bioremediation once they are low-cost and high-efficient in pollutants removal. Thus, waste products from mushroom farming such as cutting and substrate of *Lentinula edodes* (popularly known as shiitake) and *Agaricus bisporus* (also known as champignon) were evaluated as biosorbents for metallic contaminants copper (Cu) and lead (Pb). Shiitake and champignon stalks, and shiitake substrate (medium in which shiitake was cultivated) were dried, grounded, characterized and experimented to remove Cu and Pb from contaminated water. The Sips model was used to establish the adsorption isotherms. Regarding Cu, champignon stalks have the best removal efficiency (43%), followed by substrate and stalks of shiitake (37 and 30%, respectively). Pb removals were similar among three residues (from 72 to 83%), with the champignon stalks standing out. The maximum adsorption capacities (*q_max_*) for Cu in shiitake and champignon stalks were 22.7 and 31.4 mg/g^−1^, respectively. For Pb, *q_max_* for shiitake and champignon stalks, and shiitake substrate were 130.0, 87.0 and 84.0 mg/g^−1^, respectively. The surface morphology of the champignon stalks revealed an organized and continuous structure. After an interaction with metals, the stalk of champignon accumulated the metal ions into interstices. Mushroom residues showed a relevant adsorption efficiency, especially for Pb. Mushroom farming waste are a very low-cost and promising alternative for removing toxic heavy metals from aquatic environment.

## 1. Introduction

Contamination by heavy metals arises from the intense and improper disposal of electronic materials and household appliances, automobile industry, coal mining, among other sources [1]. The persistence of metals in the aquatic environment can lead to bioaccumulation, transforming the life cycle of several animals, as they can directly interfere in the enzymatic cellular processes, and morphological differentiation in different organs, such as gills, spleen, liver, and muscles [2,3]. Among metals, lead (Pb) and copper (Cu) compounds are easily found in water, such as rivers, ponds, lakes, tanks, by inappropriate discarding industrial effluents, mainly from steel mills [4].

The public health concern associated with Pb compound contamination is longstanding, but it is still a serious issue [5]. Contamination with Pb compound may be linked to the irregular/illegal disposal of waste from factories and mining, especially in undeveloped countries [6]. Some Pb compounds are highly persistent in the environment as they can be adsorbed by soil and sediments, particularly those rich in hydrous iron oxide. The chemical stability occurs as there are several Pb ligand forms, sometimes linking with another Pb, or some organic or inorganic groups, in addition to the formation of oxides [7,8]. Generally, the Pb concentration in sediments is around 30 to 45 mg/kg^−1^, but the level can reach 700 to 2600 mg/kg^−1^ in polluted rivers [9]. Lead compounds inhibit enzymatic activity, such as δ-aminolevulinic acid dehydratase (ALA-D), coproporphyrinogen oxidase, and iron chelatase, causing anemia in human beings [10]. Moreover, Pb compounds are particularly neurotoxic for children, inducing neurological damage, delayed psychomotor development, and learning difficulties [11], where it reaches a level of 10 µg/dL^−1^ Pb in the children’s bloodstream [9].

Regarding Cu, this metal is widely used in several segments, from the shape of metal alloys, oil refining, pesticides to micronutrients, involved in numerous enzyme processes [12]. However, in high concentrations, Cu induces toxic effects [13,14]. Copper concentration can vary from region to region, and they are influenced by climatic and geological factors as well as industrial and agricultural activities. Word Health Organization (WHO) guidelines recommend, for drinking water, at most 2 mg/L^−1^ [15]. In soil, the reference value in Brazil is 35 mg/kg^−1^, but in industrial regions, the concentrations can reach 10 g/kg^−1^ [16]. In a study developed in Europe, vineyards had the highest mean soil Cu concentration (about 49 mg/kg^−1^) [17]. The toxic effects are diverse, and 10 mg/day^−1^ of Cu can cause brain, kidney, and liver damage [14,15,18], in addition to accumulating in tissues such as bone [19].

Coagulation, flocculation, decantation, filtration, and disinfection are conventional methods used by most water treatment plants. However, these conventional methods have some disadvantages, such as the poor ability to remove micromaterials, the use of chemicals and the need for accurate dosing (especially in the flocculation step), frequent monitoring, trained operators, and expense [20]. Bioremediation, for both water and wastewater treatment plants, represents an advantageous alternative due to the reduction in operation steps and chemical uses, the decrease in costs, and moreover by increasing the efficiency in removing contaminants. Bioremediation can be described as a technique in which bacteria, fungi, algae or even a biomolecule or biomaterial are added to a contaminated site to attenuate, decrease, or biochemically degrade the contaminants present there [21]. Bioremediation can take place through biosorption (absorption or adsorption) and biofiltration, among others [22]. Adsorption is one of the main techniques used for decontamination of aqueous medium polluted by metals. A large variety of sorbents can be used to remove heavy metals from the aquatic environment, such as magnetic nanoadsorbents [23] and agricultural residues [24].

Among agricultural residues, those from edible mushroom waste production can be highlighted. Between 1997 and 2012, annual per capita consumption of edible mushrooms increased from about 1 to over 4 kg/y^−1^ worldwide [25]. Accordingly, in this way, the generation of their residues increased in the same direction, making stalks and substrates a possible issue, due to their disposal in the environment. Lau et al. [26] declared that 1 kg of mushrooms can generate 5 kg of spent mushroom substrate.

Mushroom production wastes can be divided into cuttings and substrate. Cuttings are the stalks of edible mushrooms not suitable for consumption as food, but their cell wall is an important source of chitosan [27]. Chitin and chitosan are compounds able to chelate metals, such as Pb [28]. The substrate is a lignocellulosic-based material where the mushrooms are cultivated, made of sawdust enriched with vegetable bran. After harvesting, the substrate is discarded into the environment [25].

Despite there being some studies that used edible mushrooms for metal bioremediation, some have applied mushroom biomass [29,30]. Moreover, bacteria and fungi consortium were also studied in removal of metals [31]. Innovatively, in this study we propose: (*i*) to produce powdered biomaterials only using mushroom residues; (*ii*) to evaluate these biomaterials from stalks of *Agaricus bisporus* (champignon) and *Lentinula edodes* (shiitake), as well as the spent mushroom substrate of shiitake as biosorbents to remove heavy metals such as lead and copper from water. The great innovation of this study is the potential use of a low-cost biomaterial, such as mushroom waste production, which is generally discarded. Indeed, these residues can be transformed into a porous biomaterial with high adsorption capacity with a few steps of processing.

## 2. Materials and Methods

In this study, we used two types of residues: the stalks of edible mushrooms not suitable as food, and the spent mushroom substrate (the leftover substrate generated by mushroom industries after harvesting period of mushroom), named here only as substrate.

Menk et al. [32] structurally and chemically characterized the point of zero charges (PZCs) of stalks of shiitake and champignon, and substrate of shiitake. PZC ranges were identified as 4.7 ± 0.2, 6.3 ± 0.2, and 4.5 ± 0.2 for stalks of shiitake and champignon, and substrate of shiitake, respectively [32]. Thus, all metal solutions were adjusted for the above specific pH values, since PCZ implies the successful pH value of the adsorption process. Regarding structural data, analyses were also run in a previous article from our research group [32]. Stalks of shiitake and champignon showed high porosity (71.3 and 70.0, respectively), compared to 67.2% porosity to substrate of shiitake. Additionally, regarding chemical characterization, the vibrational spectrum of both stalks and substrate were comparable to the chitosan spectrum [32].

### 2.1. Sample Preparation

The stalks and the substrate of shiitake were supplied by local producers from Sorocaba (São Paulo State, Brazil). The stalks of champignon were supplied by producers from Bragança Paulista (São Paulo State, Brazil). The substrate included a mixture of eucalyptus sawdust used for mushroom cultivation, containing rice and wheat bran, charcoal, and calcium carbonate (for pH correction). The mushrooms remained on this substrate for 4 to 7 months until they were ready for harvest. The stalks and substrate were oven-dried (at 50 °C for two days). Subsequently, the dried material was ground in a knife mill (Marconi^®^—MA340, #0004201) and passed through a sieve of different particle sizes. The particles size of <0.180 mm (>80 mesh) used in this study was chosen based on our previous study [32].

### 2.2. Removal Efficiency Over Time

The metals Pb (Lead (II) acetate trihydrate (99.999%, trace metals basis) and Cu (Copper (II) sulfate pentahydrate (≥98.0%) were purchased from Sigma-Aldrich (St. Louis, MO, USA). The assays were run according to Menk et al. [32]. For Cu, 0.5 g of the stalks of shiitake, champignon, or shitake substrate was added to an Erlenmeyer flask containing 60 mL of Cu solution of 1000 mg/L^−1^. Pb tests were run in the same conditions described above (Pb solution of 1000 mg/L^−1^). The metal solutions were made with bidistilled water. The concentrations are higher than those found environmentally, and these concentrations were chosen based on studies of Chen et al. and Amar et al. [33,34]. However, removal efficiency and isotherms need to be run in high concentrations in order to saturate the aqueous medium and thus be able to evaluate the bioremediation potential. The metal solutions were adjusted for specific pH values according to PZC, using NaOH or HCl solution.

Each metal was placed independently in an Erlenmeyer flask. The flasks were kept under constant agitation at 100 rpm in an orbital shaker (Nova Técnica, NT 715), orbital with 10 mm amplitude, for specified times of 10, 20, 30, 45, 60, 120, 240, 360, 720, and 1440 min at 25 °C. The temperature of 25 °C was chosen as it is considered the standard ambient temperature, at which any water treatment plant could act. At each period, samples from metal solutions were taken, and the adsorbents were removed completely from the solution, using a 1.22 µm particle retention filter. The number of samples, in total, was 20 per metal. The remaining solutions were analyzed using inductively coupled plasma mass spectrometer (ICP-MS) according to Section 2.4.

The removal efficiency of the heavy metal, here indicated as metal removal (%wt), from an aqueous solution, was calculated by Equation (1):

(1) $$Metal\;removal\;\left(\%\right)=\frac{\left(C_{0}-C_{e}\right)}{C_{0}}\times 100\;$$

where *C*_0_ is the initial concentration of the metal solution (mg/L^−1^) and *Ce* is the equilibrium concentration (mg/L^−1^), which means the final concentration of the metal in the solution at a specific time.

### 2.3. Adsorption Isotherm

The capacity of the adsorbent to retain the contaminant on its surface was assessed through assays with different concentrations of the contaminant, related to the removal efficiency over time. For this, 0.5 g of each adsorbent was added to 60 mL of different concentrations (10, 25, 100, 250, 500, 750, and 1000 mg/L^−1^) of each metal. The systems were kept under constant agitation at 100 rpm, at 25 °C, in an orbital shaker (Nova Técnica, NT 715) for 24 h. The systems were filtered according to Section 2.2 and analyzed using ICP-MS according to Section 2.4.

The adsorption equilibrium measures the concentration of a solute in an equilibrium state that can be absorbed by a tested absorbent. Therefore, the adsorption equilibrium (*q*) can be calculated as the equilibrium concentration of a solute (*C_e_*) subtracted from the initial concentration of the solute (*C*_0_) in mg/L^−1^ for a given mass of adsorbent (m) added in a volume (*V*). It is expressed in mg g^−1^ and is calculated by Equation (2):

(2) $$q=\frac{\left(C_{0}-C_{e}\right)\cdot V}{m}\;$$

The adsorption isotherm was estimated according to the Sips model, which combines the Langmuir and Freundlich models [35,36], and can be expressed by Equation (3):

(3) $$q=\frac{q_{max}\cdot K_{LF}\cdot C_{e}{}^{n}}{\left(1+K_{LF}\cdot C_{e}{}^{n}\right)}\;$$

where *q* is the amount adsorbed, *q_max_* is the maximum adsorption capacity, *K_LF_* is the equilibrium constant, *Ce* is the equilibrium concentration, and *n* is the heterogeneity parameter.

The *K_LF_*, *q_max_* and *n* parameters were estimated by OriginPro^®^ 8 software (OriginLab Corporation, Northampton, MA, USA).

### 2.4. Evaluation of Copper (Cu) and Lead (Pb) in Water

Cu and Pb concentrations in the solution were measured using an inductively coupled plasma mass spectrometer (ICP-MS) with collision cell. The equipment was operated with high-purity argon (99.999%, Praxair, São Paulo State, Brazil). The operating conditions were set according to [37]. The samples were acidified (1% *v*/*v*^−1^) with sub-boiled (DST-1000, Savillex, Eden Prairie, MN, USA) 14 M HNO_3_ (Synth, Brazil) and directly injected into the ICP-MS equipment.

Standards for calibration were prepared by diluting the commercial standards (10,000 ng/mL^−1^ acquired from Perkin Elmer, Waltham, MA, USA) in HNO_3_ 1% *v*/*v*^−1^ over the range of 0 to 100 ng/mL^−1^. The correlation coefficient (R^2^) for calibration curves was >0.9999. The performance of the method was controlled by the analysis of blanks (ultrapure water) and Standard Reference Material (SRM) 1640a Natural Water, from the National Institute of Standard and Technology.

### 2.5. Biomaterial Morphology

The mushroom surface morphology and elemental compositions in the biosorbent surfaces were, respectively, investigated using Scanning Electron Microscopy (SEM) and Energy Dispersive Spectroscopy (EDS) (JEOL, Model IT200).

This assay was carried out with the adsorbent that showed the best set of results—in this case, the champignon. Three samples were evaluated: (*i*) stalks of champignon (considered blank sample, without any contact with metal solutions); (*ii*) stalks of champignon after 20 min in contact with Cu solution; and (*iii*) stalks of champignon after 120 min in contact with Pb solution. The samples that had contact with the metal solution were oven-dried at 50 °C, for one day before being morphologically evaluated. Sample preparation for both SEM and EDS consisted of placing them on carbon tape for metallization with gold (99.9% pure).

## 3. Results and Discussion

### 3.1. Removal Efficiency over Time

**Figure 1:**
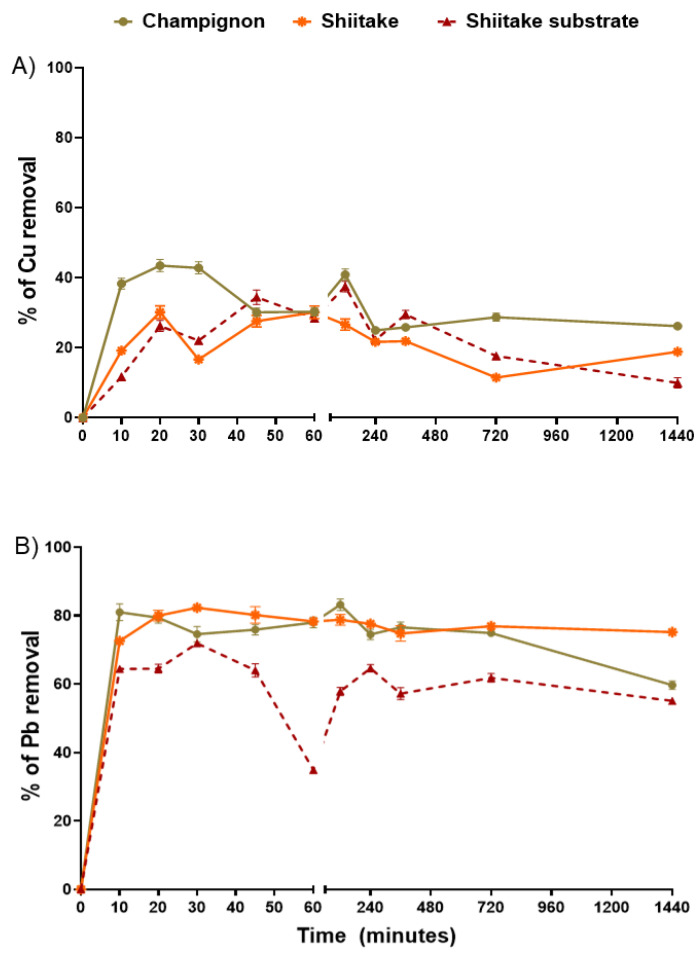
Copper (Cu) (**A**) and Lead (Pb) (**B**) removals. Both were assessed with Cu or Pb solution, 1000 mg/L^−1^, and 0.5 g of adsorbent, such as stalks of shiitake and champignon, and substrate of shitake.

This study evaluated the stalks of champignon and shiitake mushrooms and the shiitake substrate as biosorbents for Pb and Cu. The Cu removal efficiency over time is shown in Figure 1A.

The highest rates of Cu removal for shiitake substrate were 34 and 37% at 45 and 120 min, respectively, followed by desorption. Maximum removal rates for stalks of shiitake were 30% at 20 and 60 min. For stalks of champignon, Cu removal reached 43% in the first 20 min. Stability was achieved after approximately 720 min. However, for greater certainty of the stabilization of the adsorption/desorption rate of the samples, a time of 24 h was adopted for the Cu isotherm assays.

The results of Pb removal efficiency over time are shown in Figure 1B. The biosorbents showed very satisfactory results, with adsorption rates above 60%. Maximum removal rates varied from 72% for the substrate of shiitake (30 min), 82% for stalks of shiitake (30 min), and 83% for stalks of champignon (120 min). The stability was reached after about 360 min. Therefore, Pb isotherm assays were evaluated for 24 h. Pb removal efficiency was much more effective in stalks than in substrate. Results from our previous research, regarding microtomography, can explain these results, since shiitake and champignon stalks had better open pore and porosity % in comparison to the substrate [32].

Liu et al. evaluated the capacity of *Pleurotus cornucopiae* mushroom in removing two different metals (i.e., Cu and Cadmium—Cd) from substrates naturally contaminated with those metals. Substrates were evaluated after the growth of the mushrooms, and the results showed that *P. cornucopiae* was able to reach 36 and 22% of Cu and Cd retention, respectively [38]. Data for Cu removal were similar to the results found for the adsorbents evaluated by our group.

Comparing mushroom residues with other adsorbents, El-Ashtoukhy et al. evaluated the removal of Cu and Pb by pomegranate peel, reaching adsorbent saturation after 120 min [39]. These authors found the adsorption of Pb in raw pomegranate peel reached 90%, similar to those obtained with the stalk of champignon, while for Cu they found 98% removal. In another interesting study, it was observed that the removal capacity by *Penicillium simplicissimum* after 15 min is constant for Pb, reaching an adsorption of 56.6% [33]. Amar et al. evaluated the adsorption of metals (Pb and Cu) for olive stones, and the maximum removal rate was 1 h, with 75.2 and 40%, respectively [34]. In another study, Amarasinghe and Williams used tea waste as a sorbent and they achieved adsorption rates of 94 and 70% for Pb and Cu, respectively [40].

On the other hand, comparing the metal removal efficiency, it is important to realize a common point in all the studies reported, regardless of the type of biosorbent: the Cu biosorption is always less than other metals. In this way, we could suggest that the biosorbents, including the mushroom residues, are more easily saturated with this metal (i.e., Cu). Thus, it may be necessary to use a larger mass of biosorbent for this type of contaminant.

### 3.2. Adsorption Isotherm

**Figure 2:**
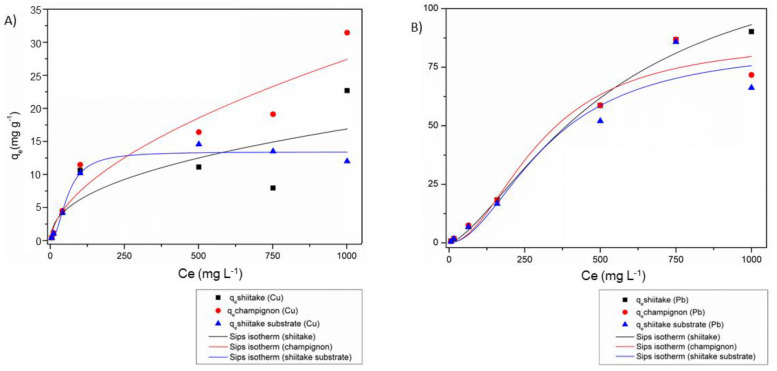
Sips theoretical isotherms to Copper (Cu) (**A**) and Lead (Pb) (**B**) by stalks of shiitake, stalks of champignon, and substrate of shitake. *C_e_* and *q_e_* correspond to the equilibrium concentration (mg/L^−1^) and the amount adsorbed in equilibrium (mg/g^−1^), respectively.

Sips theoretical isothermal model was applied to the three adsorbents, and results for Cu and Pb are depicted in Figure 2A,B, respectively.

The summary comparison between Pb and Cu isotherms is presented in Table 1. The Sips model did not fit adequately for Cu in the stalk of shitake, since the R^2^ value was very low (0.546). The Sips model is the union of Langmuir and Freundlich models, and these models were evaluated individually, but they did not fit adequately (data not showed). We suppose that the concentrations of Cu solution used were not suitable, and it could be a limitation of the method. On the other hand, for the Pb, the Sips model presented an excellent fit to all adsorbents (i.e., with R^2^ values > 0.919).

The highest adsorption capacity (*q_max_*) for Cu occurred at 1000 mg L^−1^ for stalks of champignon (31.44 mg g^−1^) followed by stalks of shiitake (22.68 mg/g^−1^). On the other hand, for the substrate of shiitake, the highest *q_max_* (14.57 mg/g^−1^) was obtained at 500 mg/L^−1^.

The equilibrium constants (*K*) for Cu are also presented in Table 1. These results express the ratio between the adsorption and desorption constants over time. Li et al. evaluated the removal of Cu, Zinc, and Mercury from water by inedible stipe of mushrooms [41]. They used different mushroom species from ours and demonstrated that *Auricularia polytricha* had *K* values of 0.2893 and 0.0510 by the Freundlich and Langmuir models, respectively. *K* is a factor influenced by several aspects, such as source of biomaterial (i.e., type of species), physiological age, and tissue morphology [42]. Thus, once we used different species, a variation in *K* values can occur.

The empirical parameter of system heterogeneity (*n*) determines that the Sips equation will be reduced to a Langmuir equation if *n* ≥ 1, or the Freundlich equation if *n* ≤ 1 [36,43,44]. Thus, for Cu, shitake and champignon stalks indicated a better affinity to the Freundlich equation, whereas for shiitake substrate the *n* value indicated a better fit with the Langmuir isotherm.

The best Pb *q_max_* occurred for the initial concentration of 1000 mg/L^−1^ for the stalk of shitake (90.18 mg/g^−1^), while for the stalk of champignon and substrate of shiitake (both with a concentration of 750 mg/L^−1^), the best experimental values were 86.74 and 8.79 mg/g^−1^, respectively. Comparatively, the *q_max_* obtained by the Sips equation was similar to the experimental values of 130.0 mg/g^−1^ for shiitake stalks, 87.0 mg/g^−1^ for champignon stalks, and 84.0 mg/g^−1^ for shiitake substrate. The *n* values for Pb indicated a better experimental affinity to the Langmuir equation (i.e., *n* ≥ 1).

In study about soil bioremediation, champignon was studied as an adsorbent for Pb and Cd [45]. The authors achieved isotherm results akin to the present study; however, for Pb, the R^2^ obtained for the Langmuir model was 0.960 and the *q_max_* was 19.7 mg/g^−1^. Similar data were found in a study using four samples: raw pomegranate peel, activated carbon prepared from pomegranate peel (AC1), activated carbon prepared from chemically treated (phosphoric acid and zinc chloride 1:1 solution) pomegranate peel (AC2) and finally activated carbon prepared from chemically treated (n nitric acid 10%wt solution) pomegranate peel (AC3). The best R^2^ for Pb, explained by the hydration enthalpy, was R^2^ of 0.9994 in the raw sample, followed by AC1 (0.9989), AC2 (0.9978), and AC3 (0.9994), indicating the occurrence of a higher activity of Pb cations than Cu cations for adsorbents [39]. In our case, the obtained R^2^ values, according to the Sips model for Pb, were 0.953 for stalks of champignon, 0.991 for stalk of shiitake, and 0.919 for substrate of shiitake.

### 3.3. Scanning Electron Microscope (SEM)

**Figure 3:**
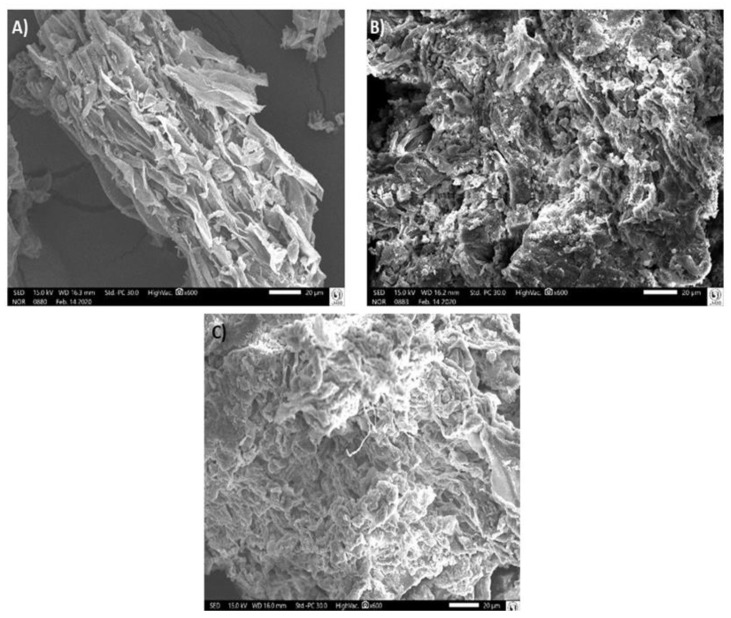
Scanning electron microscopy (SEM) of particles from stalks of champignon (**A**); stalks of champignon after 20 min of contact with Cu (**B**); and stalks of champignon after 120 min in contact with Pb (**C**). Magnification of 600×.

By using SEM, the adsorbent with the best result set for adsorptive capacity was evaluated. The stalk of champignon was chosen as the best adsorbent, considering both the removal efficiency and the isotherm. Thus, the powder from stalks of champignon was evaluated before contact with the metal solutions (considered blank sample, Figure 3A), and after 20 and 120 min in contact with Cu and Pb, respectively, Figure 3B,C.

Figure 3A shows that the stalks of champignon particles, without metal interaction (only oven-dried at 50 °C, grounded and sieved), presented natural and continuous structures, with smoother and more organized aspects. This structure could provide a large surface area and facilitate mass retention, contributing to the adsorption process. The images corresponding to the particles of the champignon stalks in contact with the metals (Figure 3B,C) presented an accumulation of these metallic ions, resembling more coiled structures with residues in their interstices. Qiao et al. reported structural changes in the *L. edodes* mushroom surface after adsorption of Cd. According to these authors, chemical interactions between functional groups such as hydroxyl groups would be responsible for the marked changes [46]. We also suggested that the metals (Cu and Pb) could change the functional groups from multimer to monomers, increasing the number of exposed functional groups and consequently the absorbent area. Moreover, it is important to consider the metal solutions in our study, which had their pH values adjusted according to PCZ. We propose the adjustment implies the success of the adsorption process by arranging the structure of particles, facilitating the interactions between metals and adsorbent.

**Figure 4:**
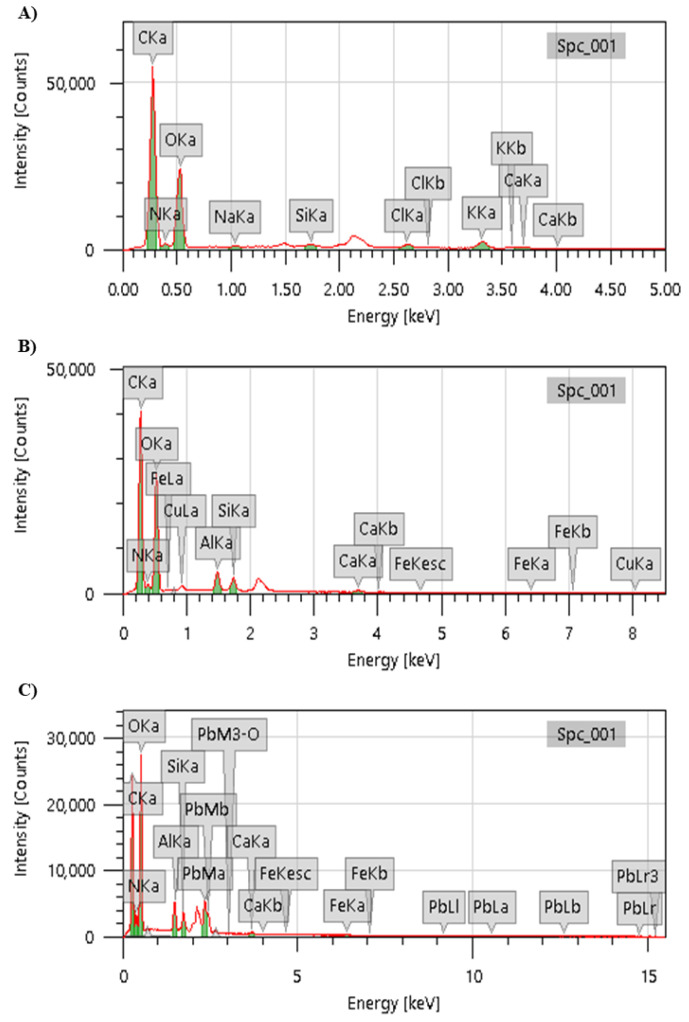
Energy dispersive spectroscopy (EDS) spectrums separated into peaks of the capital letters K, L or M indicate the shell containing the chemical element, whereas the greek letters (a = alfa, b = beta, r = ro, M = mi) indicate energy in Kev; in ground champignon (**A**), champignon after 20 min of contact with Cu (**B**), and after 120 min in contact with Pb (**C**).

The elementary chemical analysis carried out by EDS showed that the champignon stalks did not present any mass percentage of Cu and Pb (Figure 4A) before contact. On the other hand, the mass percentages of Cu and Pb found in the particles after contact with the elements were 2.51 ± 0.06 and 14.98 ± 0.09 wt% (Figure 4B,C, respectively). These findings corroborate the removal efficiency and isotherm data. For example, the presence of chitosan was evidenced as a component of the champignon, considering the characterization of chemical groups performed by Menk et al. in the champignon stalk [32]. In this sense, hydroxyl, carboxyl, and amide groups indicated that polysaccharides, as well as proteins present on mushrooms [46], can bind metals through chemical interactions, facilitating the adsorption process.

## 4. Conclusions

Mushroom residues are promising biomaterials with favorable properties for adsorption of a wide range of environmental contaminants. In this study, stalks and substrates from two types of mushrooms showed to be an appropriate matrix for metal adsorption for contaminated water. For Pb, the Sips model proved to be satisfactory, with a very good adsorption capacity for the three biosorbents. This work also demonstrated that stalk of champignon was the best candidate for the adsorption process, considering both the removal efficiency and the isotherm model. The biomaterials proposed in this work are versatile, non-toxic, and they can represent an ecofriendly alternative for biosorbents, once they are easily accessible and there is no need for any synthetic route for its manufacture. More specifically, we could suggest the powder of the stalk of champignon has the potential to be used directly in water treatment plants, with subsequent removal by a simple filtration process, or even as packing fixed-bed columns.

## Figures and Tables

**Table 1:** A general summary of the isotherm model parameters according to the Sips model and comparing the tree biosorbents evaluated. The *K* and *q_max_* parameters correspond to the equilibrium constant and maximum adsorption capacity, respectively. The heterogeneity is represented by *n*.

Parameters	Stalk of Champignon	Stalk of Shiitake	Substrate of Shiitake
	Copper (Cu)		
R^2^	0.889	0.546	0.970
*K* (L/mg^−1^)	3.21 × 10^−5^	8.82 × 10^−5^	2.6 × 10^−4^
*q_max_* (mg/g^−1^)	31.44	22.68	14.57
*n*	0.56	0.43	2.04
	Lead (Pb)		
R^2^	0.953	0.991	0.919
*K* (L/mg^−1^)	1.40 × 10^−5^	9.56 × 10^−5^	1.48 × 10^−5^
*q_max_* (mg/g^−1^)	86.74	90.18	8.79
*n*	1.95	1.47	1.92

## Data Availability

Data and publication materials are available from the corresponding author upon reasonable request.
